# High-Content Imaging for Large-Scale Detection of Low-Affinity Extracellular Protein Interactions

**DOI:** 10.1177/2472555219879053

**Published:** 2019-10-03

**Authors:** Laura Wood, Gavin J. Wright

**Affiliations:** 1 Cell Surface Signalling Laboratory, Wellcome Trust Sanger Institute, Cambridge, UK

**Keywords:** high-content imaging, ligand–receptor interactions, extracellular, high-throughput

## Abstract

Extracellular protein interactions coordinate cellular responses with their local environment and have important roles in pathogen invasion and disease. Due to technical challenges associated with studying binding events at the cell surface, the systematic and reliable identification of novel ligand–receptor pairs remains difficult. Here, we describe the development of a cell-based assay using large-scale transient transfections and high-content imaging (HCI) to detect extracellular binding events. We optimized the parameters for efficient transfection of human cells with cDNA plasmids encoding full-length cell surface receptors in 384-well plates. Using a range of well-characterized structurally diverse low-affinity cell surface interactions, we show that transfected cells probed with highly avid ligands can be used to successfully identify ligand–receptor pairs using an HCI platform and automated image analysis software. To establish the high-throughput potential of this approach, we also screened a pool of ligands against a collection of 2455 cell surface expression clones and found that known ligand–receptor interactions could be robustly and consistently detected across the library using this technology.

## Introduction

Cell surface receptors play an important role in sensing the local environment and transducing this information to the cell interior where signaling responses can be appropriately controlled and coordinated. A large portion of Food and Drug Administration (FDA)-approved drugs target cell surface proteins, and future treatments that focus on blocking ligand–receptor binding events may have important implications in preventing disease and pathogen infections.^1^ Membrane-spanning receptors, however, are notoriously difficult to study, as the absence of a plasma membrane can lead to solubility issues and changes in native structure and function.^2^ In addition to this, extracellular binding events are typically low affinity (*K*_D_ in the micromolar to millimolar range) and there are several thousand known genes encoding cell surface receptors in the human genome.^3–7^ There is therefore a need for assays that take account of the biochemical difficulties while also incorporating high-throughput elements to ensure efficient screening.^8^

One large-scale biochemical approach to identify low-affinity extracellular protein interactions involves testing for direct binding events between soluble recombinant proteins that comprise the ectodomain regions of cell surface receptors. To increase binding avidity, these proteins are fused with domains that promote multimerization and can be systematically screened against one another in a simple plate-based format or by using microarray technology to spot ectodomains in a defined pattern onto slides. These methods have successfully identified extracellular protein binding events in many biological contexts, including pathogen–host cell interactions.^9–11^ Screening of recombinant proteins is resource-intensive and library design is often restricted to receptors that have a single contiguous region exposed on the external-facing surfaces of the cell. This means that many multipass membrane proteins and multisubunit complexes are not generally suitable for this approach.

The use of cell-based assays provides an opportunity to overcome some of these challenges, allowing receptors to be studied within the context of the cell surface microenvironment. For example, mass spectrometry-based techniques have been used on living cells where probes with chemically derived tags are able to covalently capture and purify endogenous receptors^12,13^ and CRISPR/Cas9 technology can generate genome-wide libraries of knockout cells that can be sorted by simple readouts, such as a loss of pathogen invasion^14^ or a reduction in the binding of a soluble recombinant ectodomain.^15^ CRISPR/Cas9-based tools can also be used in the gain of binding studies where the transcriptional activation of endogenous genes (CRISPRa) has been employed to overexpress all cell surface proteins in the human genome, successfully identifying receptors bound by monoclonal antibodies and highly avid ligands.^16^

As an alternative gain of binding approach, transient transfection of cDNAs encoding full-length receptors can promote receptor overexpression on the surface of cells. More classical approaches using expression libraries generated from cell/tissue sources have been very successful, but iterative rounds of selection and screening on complex pools decrease throughput.^17^ Therefore, more high-throughput implementations of this approach are required, as shown by the recent commercialization of a cell microarray, where expression plasmids spotted onto slides and reverse transfected into cells are used to identify receptors bound by a labeled probe.^18,19^ Here, we aimed to set up a cell-based assay where cDNA-induced overexpression of cell surface receptors could be used to screen for extracellular interactions in 384-well plates with high-content imaging (HCI) and automated image analysis software. Recombinant ectodomains screened against transiently transfected cells were pentamerized to increase the binding avidity of potentially weak cell surface interactions and GripTite HEK293 cells were used to ensure adherence following multiple wash steps in immunofluorescence procedures. We implemented this approach in a high-throughput screening format using a collection of 2455 human cell surface expression clones and found that known ligand–receptor interactions were detected efficiently across the library using this technology. Importantly, this method provides a platform to study biochemically challenging receptors within the context of an intact plasma membrane.

## Materials and Methods

### Recombinant Protein Production and Normalization

The ectodomain regions of extracellular proteins were codon-optimized for expression in human cells and synthesized with flanking *Not*I (5′) and *Asc*I (3′) restriction sites (GeneArt, ThermoFisher, Waltham, MA). Ectodomain sequences were subsequently cloned into mammalian expression plasmids containing C-terminal tags (Cd4d3+4-COMP-blac-3xFLAG-6xHis).^20^ Regions 3 and 4 of rat Cd4 were used as an antigenic sequence, and a cartilage oligomeric matrix protein (COMP) peptide was used to pentamerize ectodomains, producing highly avid protein complexes. The β-lactamase enzyme and the 6xHis-tag were used for normalization and purification, respectively. All ectodomains were expressed with their endogenous signal peptide sequences, except LPHN1 and GPR64, which were designed to include an exogenous signal peptide.^16,21^

Recombinant proteins were produced as soluble secreted ectodomains by transiently transfecting HEK293 cells as described previously.^22,23^ Briefly, HEK293E cells grown in FreeStyle media (Gibco, ThermoFisher, Waltham, MA) supplemented with 50 µg/mL G418 and 1% (v/v) heat-inactivated fetal bovine serum (FBS; Sigma, St Louis, MO) were prepared in 100 mL suspensions at a density of 2.5 × 10^5^ cells/mL. After 24 h, cells were transiently transfected and cultured for 6 days before supernatants were harvested and filtered through a 0.2 μm filter. Supernatants were either concentrated with a 20 k MWCO spin concentrator (Sartorius, Gottingen, Germany) or, for His-tag purifications, passed through a HisTrap HP column on an AKTApure (GE Healthcare, Chicago, IL). Purified proteins were buffer exchanged into phosphate-buffered saline (PBS) using PD midiTrap G-25 columns (GE Healthcare) and stored at 4 °C with 2 mM sodium azide. Recombinant protein ectodomains were normalized using β-lactamase enzyme activity assays through the hydrolysis of the colorimetric β-lactamase substrate nitrocefin.^23^ In brief, 30 μL of serially diluted supernatants, or purified proteins, was incubated with 60 μL of 125 μg/mL nitrocefin at room temperature for 20 min. The rate of nitrocefin hydrolysis was measured at an absorbance of 485 nm with a Spark microplate reader (Tecan, Mannedorf, Switzerland).

### Antibody Production, Purification, and Fluorescent Labeling

The hybidroma cell line OX68 (ECACC 94011007) secretes a mouse IgG2a monoclonal antibody that recognizes domains 3 and 4 of rat Cd4. Hybridomas were adapted to serum-free media (Hybridoma-SFM; Gibco) and the supernatant harvested and filtered through a 0.2 μm filter. The OX68 antibody was purified with a 5 mL HiTrap Protein G HP column using 20 mM sodium phosphate, pH 7.0 (binding buffer), and 0.1 M glycine, pH 2.7 (elution buffer), on an AktaExpress (GE Healthcare). Eluted fractions of 500 µL were collected in 96-deep-well plates containing 40 µL of 1 M Tris, pH 9.0, to neutralize solutions. Fractions were subsequently dialyzed against PBS and stored at 4 °C before labeling. The OX68 antibody was labeled with a 20× molar excess of Alexa Fluor 488 NHS Ester (Invitrogen Molecular Probes, Carlsbad, CA) in 0.1 M sodium bicarbonate, pH 8.5, for 1 h at room temperature. Reactions were quenched at a final concentration of 0.1 M Tris, pH 8, for 5 min at room temperature and immediately dialyzed against PBS. A preservative of 2 mM sodium azide was added to fluorescently labeled antibodies and aliquots frozen at –20 °C.

### cDNA Library Storage and Plasmid Purification

A collection of expression plasmids encoding full-length cell surface receptors were purchased from OriGene Technologies (Rockville, MD) and GeneCopoeia (Rockville, MD) and stored as bacterial glycerol stocks (**Suppl. Table S1**). Origene clones were a mixture of TrueClone untagged cDNA clones derived from human cDNA libraries, TrueORF tagged ORF clones (Myc-DDK tag), and untagged ORF clones synthesized by the company. GeneCopeia provided expression-ready untagged ORF clones. All ORF clones are sequence verified by their respective companies. Origene’s TrueClones are assessed for the completeness of the open reading frame and compared with an associated reference. Our aim was to accumulate cDNAs encoding the longest isoforms. Competent *Escherichia coli* were produced in-house using the Inoue method from library efficiency DH5α cells (Invitrogen, Carlsbad, CA).^24^ The creation of bacterial stocks was adapted from an automated approach to DNA library preparation.^25^ Briefly, competent cells were thawed and 20 µL was distributed into each well of a 96-well PCR plate (Thermo Fisher Scientific, Waltham, MA). While on ice, 40–60 ng of plasmid DNA was added to each well and incubated for 30 min, heat-shocked for 1 min at 42 °C, and then placed back on ice for a further 2 min. For cells transformed with plasmids containing an ampicillin-resistant gene, 5 µL was directly transferred to an 8-well agar plate supplemented with appropriate antibiotics. Heat-shocked cells transformed with a kanamycin-resistant plasmid were incubated with 200 µL of TB buffer at 37 °C and plated 3 h later. Single colonies were picked and added to 96-deep-well dishes containing 1.5 mL of TB buffer and incubated for a further 18–20 h at 37 °C. Bacterial cultures were stored in barcoded 0.3 mL FluidX tubes (Brooks Life Sciences, Manchester, UK) at –80 °C at a final concentration of 40% glycerol.

To purify plasmid DNA, glycerol stocks were thawed and 5 µL distributed to 4× 24-deep-well plates containing LB media with appropriate antibiotics and incubated overnight at 37 °C. A QIAVac 96 vacuum manifold and QIAprep 96 filter plates were used to miniprep DNA in accordance with the manufacturer’s instructions (Qiagen, Hilden, Germany). The only difference was that 4× 24-well plates were centrifuged for 50 min at high speed after the addition of neutralization buffer to pellet the flock, enabling supernatants to be effectively distributed into the QIAprep 96 filter plate. The elution step was also performed twice with 100 µL of EB buffer. Concentrations ranged from ~50 to 300 µg/mL and multiple freeze–thaws of plasmid DNA were avoided.

### Cell Culture and Transfections

GripTite HEK293 cells (Invitrogen) were cultured in DMEM+GlutaMAX-I (Gibco) containing 10% (v/v) heat-inactivated FBS (Sigma), 500 µg/mL G418, and 100 µM nonessential amino acids (Gibco) at 37 °C in a humidified atmosphere of 5% CO_2_. To increase cell adherence, black-walled TC-treated 384-well plates (Corning, New York, NY) were incubated for 1 h with 25 µL of a 25 µg/mL PEImax 40K solution (pH 7) (Polysciences, Inc., Warrington, PA).^26^ To remove PEImax from the wells, plates were centrifuged upside down at 1500 rpm and left to dry under the tissue culture hood. GripTite cells at a confluency of 50%–80% were detached from culture flasks in accordance with the manufacturer’s instructions and diluted into complete media at a concentration of 2 × 10^5^ cells/mL. An automatic pipette was used to distribute 50 µL of cell suspension into each well (10,000 cells) and plates were centrifuged for 2 min at 100 rcf before being placed back at 37 °C for 24 h. Lipid-based transfections in a 384-well format were performed with a Viaflo 384 (Integra, Plainsboro, NJ) using a channel pipetting head capable of handling 0.5–12.5 µL. Two 384-well plates were prepared: a DNA plate (plate 1) and a transfection reagent plate (plate 2). To account for dead volume, a 1.5× volume reaction was created for each well. In plate 1, plasmid DNA was transferred from a stock cDNA library plate and mixed 1:1 with Optimem+GlutaMax-I (Gibco) (3.75 µL total). A master mix of Optimem+Glutamax-I and Lipofectamine 2000 (Invitrogen) was aliquoted into plate 2 (scale-up from single reaction: 2.5 µL Optimem + 0.15 µL transfection reagent). The Viaflo 384 was used to transfer 3.75 µL of transfection reagent from plate 2 into plate 1 and programmed to gently mix solutions six times, at a volume of 4 µL. This process can be efficiently repeated for multiple cDNA plates. After 20 min at room temperature, 5 µL of the cDNA/transfection mix was added to cells simultaneously using the 384-channel pipette. Plates were covered with a gas-permeable seal, placed back in the 37 °C incubator, and left for 40–48 h before fixation and staining protocols.

### Immunofluorescence Fixation and Staining

For each step, an automatic pipette was used to add liquid to 384-well plates, while multichannel aspirations were used to remove liquid. So as not to disrupt the cells, ~20–25 µL was left in the bottom of wells after each aspiration. Transfected cells incubated for 40–48 h in 384-well plates were collected from the 37 °C incubator and excess media aspirated from the wells. Supernatants containing recombinant ectodomains, or purified proteins diluted into DMEM+GlutaMAX-I, were preheated to 37 °C and a volume of 25 µL was added to each well (as 20–25 µL remains in the wells, recombinant proteins were diluted 1:1 with conditioned media). Plates were centrifuged for 2 min at 100 rcf and placed back in the 37 °C incubator for 2 h. Plates were then washed two times with 50 µL of PBS that had been prewarmed to 37°C, followed by fixation with 25 µL of 8% paraformaldehyde/PBS (Alfa Aeser, Haverhill, MA) for 20 min at room temperature (final concentration, ~4% paraformaldehyde). Cells were immediately washed two times with 50 µL of PBS and incubated with 25 µL of 1% bovine serum albumin (BSA)/PBS (diluted from aseptic 30% BSA; Sigma) containing 6.25 µg/mL Alexa488-labeled OX68 antibody and 5 µg/mL Hoechst-33342 (Invitrogen Molecular Probes) for 1.5 h at room temperature (final concentrations, ~3.125 and 2.5 µg/mL, respectively). The antibody incubation was followed by three PBS washes and plates were stored in the refrigerator protected from light until the images were ready to be acquired.

### High-Content Imaging and Analysis

The Cellomics Arrayscan VTI HCS Reader (Thermo Fisher Scientific) was used as a high-content screening platform to image 384-well plates. For each well, four fields of view in two fluorescent channels (Hoechst-33342 and Alexa488) were sequentially acquired using the 20× objective and BGRFR filter sets (BGRFR_386_23 and BGRFR_485_20). We found that using a higher magnification objective and capturing a section of the well (4 out of a possible 25 fields) worked best for the detection of cells while enabling efficient data acquisition timings. In channel 1 (Ch1), Hoechst-33342 staining was used to visualize cell nuclei, while Alexa488 detection in channel 2 (Ch2) was used to identify extracellular ligand–receptor binding events. The Cell Health Profiling BioApplication within the HCS Studio Cell Analysis Software was used for all downstream analysis. Images in Ch1 and Ch2 were preprocessed for the removal of background fluorescence. Hoechst-stained nuclei in Ch1 were segmented and defined as primary objects and a region of interest was used to capture signals in Ch2 across the whole cell. A fixed threshold in Ch2 was also applied so that only high-intensity Alexa488 signals were used for target identification. The aim was to calculate the percentage of cells that possess Ch2 target average intensity readings above a manually defined response limit. Well features were recorded and represent population statistics for all cells selected for analysis. Heatmaps of the data were created using R (www.r-project.org) and RStudio (www.rstudio.com).

## Results

### Optimization of High-Content Imaging and Automated Image Analysis to Identify Cell Surface Interactions in a 384-Well Format

**Figure 1.**
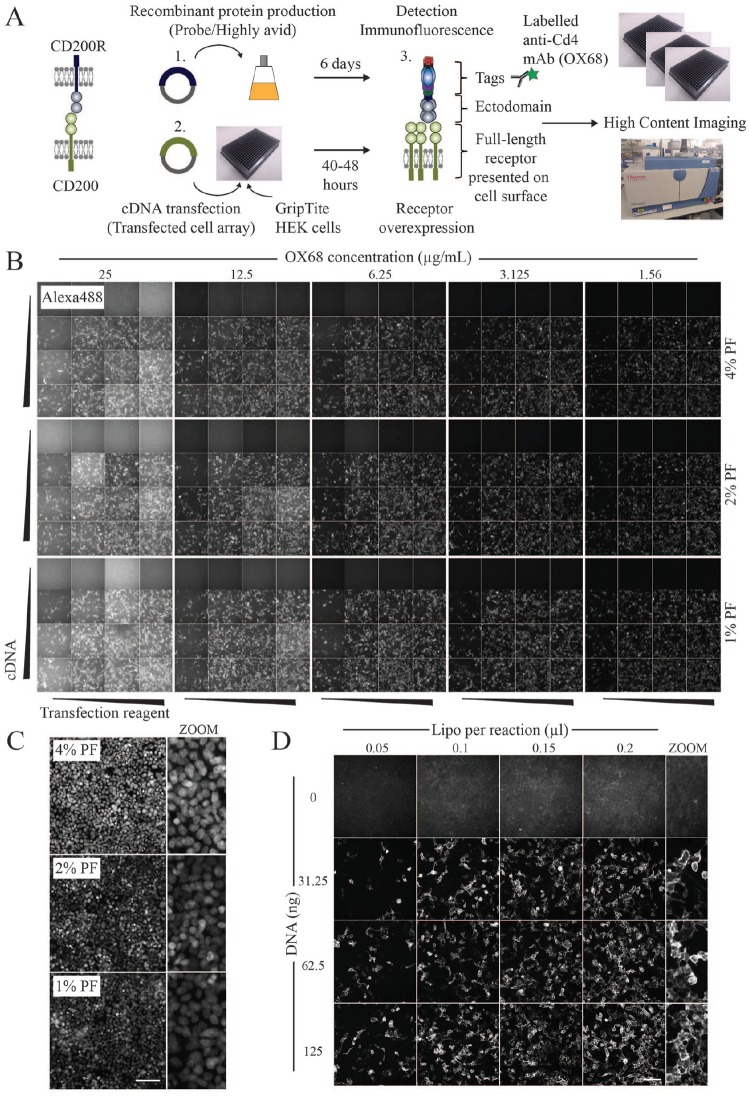
Optimization of a 384-well extracellular interaction assay using HCI. (**A**) Schematic of the general workflow for detecting extracellular receptor–ligand binding using CD200–CD200R as a model interaction. (1) A plasmid encoding the CD200R ectodomain regions in-frame with a C-terminal Cd4 tag was transiently transfected into HEK293E cells, and 6 days later recombinant proteins were collected. The ectodomains were expressed with a peptide tag that promotes pentamer formation to increase the binding avidity of weak interactions. (2) Adherent GripTite cells plated in a 384-well format were transfected with cDNAs encoding full-length CD200. (3) After 40–48 h, cells overexpressing CD200 on their cell surface were probed with CD200R proteins. Binding was detected using an Alexa488-labeled antibody (OX68) that recognizes the rat Cd4 domains 3 + 4 (Cd4d3+4) tag fused to CD200R ectodomains. Images of individual wells in a 384-well plate were acquired using an HCI system. (**B**) Testing of multiple parameters for the immunodetection of extracellular interactions. Areas of 4 × 4 wells were treated with increasing concentrations of Lipofectamine and CD200 encoding cDNA. Cells were fixed with different paraformaldehyde concentrations as indicated and stained with serially diluted Alexa488-labeled OX68 antibody. (**C**) The effect of paraformaldehyde fixation on the detection of nuclei. Acquired images of Hoechst-33342 stained nuclei in cell populations fixed with 1%, 2%, and 4% paraformaldehyde. Scale bar = 100 µm. (**D**) Alexa488-labeled antibodies can be used to detect recombinant protein binding to transfected cell populations. The amounts of cDNA and Lipofectamine (Lipo) per transfection reaction are indicated. Zoomed-in regions depict surface staining of cells. Scale bar = 100 µm.

Development of the HCI approach to study extracellular protein interactions required the optimization of experimental procedures and image acquisition protocols across 384-well plates. The general workflow is summarized in **Figure 1A**, where the interaction between CD200 and the CD200 receptor (CD200R) was used as a model low-affinity receptor–ligand interaction to establish experimental parameters for a cell-based interaction assay.^27^ We wanted to test multiple conditions that would be important for the immunodetection of extracellular interactions, including cell type, transfection efficiency, cell fixation, and antibody concentration.

For the transfection array, expression plasmids encoding full-length CD200 were complexed with Lipofectamine and distributed simultaneously into wells using a 384-channel pipette. Transiently transfected cells overexpressing cell surface CD200 were subsequently tested for binding with a highly avid probe containing the ectodomain region of CD200R. Importantly, the ectodomain is fused with a pentamerization domain that increases the binding avidity of typically weak extracellular interactions to facilitate their detection.^2,23^ To minimize the disruption of epitopes, fixation steps were performed after the addition of avid ectodomains, and permeabilization/detergent-containing wash steps were also excluded from the protocol to maintain the integrity of the plasma membrane. To assess adherence, cell nuclei were stained with Hoechst-33342, and to detect extracellular binding events, we used the anti-Cd4 monoclonal antibody (OX68) that recognizes the Cd4 tag on the recombinant probe. Images from two fluorescence channels were acquired using the Arrayscan-VTI HCI system.

We established that GripTite HEK293 cells were an ideal cell line for assay development as they combined high rates of transfection efficiency, while being sufficiently adherent to withstand multiple plate washing steps. By manually inspecting images across the 384-well plate, we determined that ~3.125 µg/mL OX68-Alexa488 could effectively detect cell surface interactions across multiple wells while exhibiting low levels of background fluorescence (**Fig. 1B**). Three concentrations of fixative were tested, and brighter, more consistent fluorescence signals in Hoechst-33342-labeled nuclei were observed with 4% paraformaldehyde (**Fig. 1C**). To establish optimal transfection conditions, we used varying cDNA–Lipofectamine ratios and found that transfection reagent volumes between 0.1 and 0.2 µL and cDNA concentrations between ~30 and 125 ng resulted in high numbers of Alexa488-positive cells (**Fig. 1D**). Importantly, ectodomain binding was absent in mock-transfected cells, while fluorescence signals in populations of cells overexpressing CD200 localized to the plasma membrane (**Fig. 1D**).

**Figure 2.**
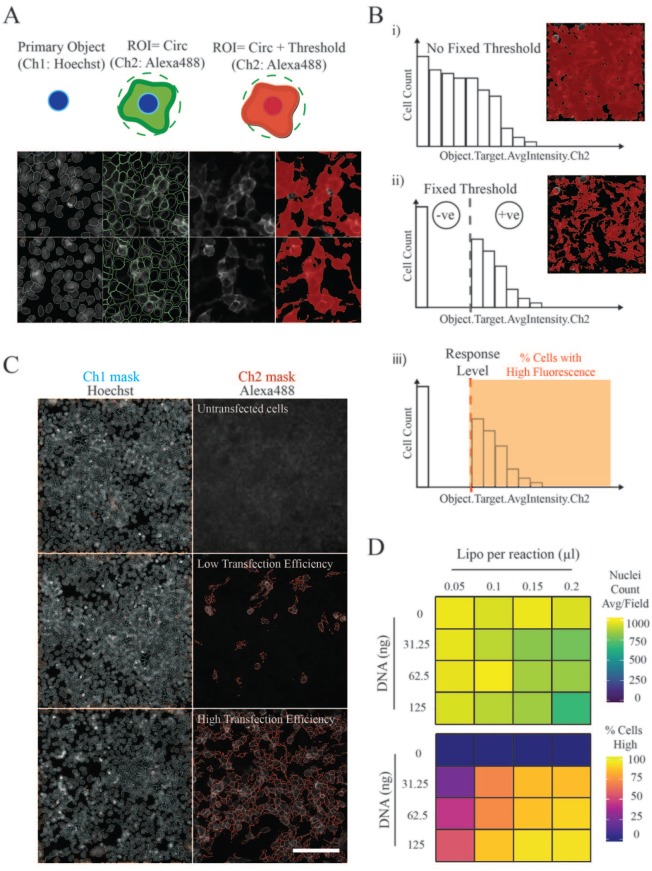
Extracellular interactions can be identified using automated image analysis tools. (**A**) Summary of image processing workflow. Hoechst-stained nuclei in channel 1 (Ch1) were segmented and defined as primary objects (cyan). In channel 2 (Ch2), a circular (Circ) region of interest (ROI) was created, extending out from the primary object to include an area covered by the whole cell (green). A fixed fluorescence threshold was set to further define the cellular area used for Ch2 Alexa488 measurements, so that only pixels above this intensity were kept for analysis and included in the target identification mask (red). (**B**) Measuring the percentage of cells in a population bound by recombinant probes. (i) When no Alexa488 fluorescence threshold is defined, all pixels within the ROI were included in the target identification mask and used for analysis. (ii) Defining the fixed fluorescence threshold allows cells to be categorized into two populations: those that were not covered by the Ch2 mask (–ve = 0), and those that were covered and therefore have an average pixel intensity equal to or greater than the defined threshold (+ve). (iii) A response level was then set to calculate the percentage of cells in the population characterized by high Alexa488 signals. (**C**) Example segmentation and target identification masks. Images of CD200–CD200R extracellular interactions overlaid with masks for the segmentation of Hoechst-stained nuclei (Ch1: cyan) and Alexa488 target identification (Ch2: red). Scale bar = 100 µm. (**D**) Image-based analysis detected variations in transfection efficiencies between wells. Heatmaps represent the average number of nuclei counted per field (i.e., primary objects) and the percentage of cells bound by CD200R ectodomains based on the automated detection of high Alexa488 signals (% Cells High). Cells were transfected with different concentrations of lipofectamine (Lipo) and CD200 cDNA.

With these optimized conditions established, we sought to set up an automated image-based analysis with the ultimate aim of increasing the scale of detection. To identify the percentage of cells in a well that had gained the ability to bind an avid probe, two fluorescence channels were acquired and analyzed simultaneously using an integrated workflow (Cell Health Profiling BioApplication; HCS Studio). Images acquired in the Hoechst channel were used to segment and mark the boundaries of individual nuclei (primary objects) (**Fig. 2A**). Nuclear objects were then used to define a region of interest surrounding the cell, and within this area, a fixed fluorescence threshold was set to detect and measure high-intensity Alexa488 signals (**Fig. 2A**). By setting a cellular response limit, the percentage of cells characterized by strong OX68-Alexa488 antibody staining could then be calculated (**Fig. 2B**). By defining this cellular state, it is therefore possible to separate ligand-bound and unbound cell populations. An important parameter dictated by transfection efficiency was the requirement to plate cells at ~50%–60% confluency, transfect, and wait 48 h before ectodomain screening. We imaged wells at high magnification to try to accurately segment nuclei and found that a large proportion of nuclei can be successfully separated, as shown by the image segmentation mask in channel 1 (**Fig. 2C**). We did observe some over- and undersegmentation across larger data sets, especially in overgrown regions that were challenging to segment, but using heatmaps for comparison, we found that nuclei count remained relatively consistent between wells (**Fig. 2D**); as a consequence, cell number is estimated, rather than absolute. The target mask in channel 2 is able to detect ligand-bound cells, even when transfection efficiencies are poor (**Fig. 2C**). We used this approach to show how varying the amount of plasmid DNA and transfection reagent affected signal readouts, which were transformed into heatmaps to facilitate analysis (**Fig. 2D**).

### High-Content Imaging Can Be Used to Identify Low-Affinity Ligand–Receptor Interactions between Different Architectural Classes of Receptor

**Figure 3.**
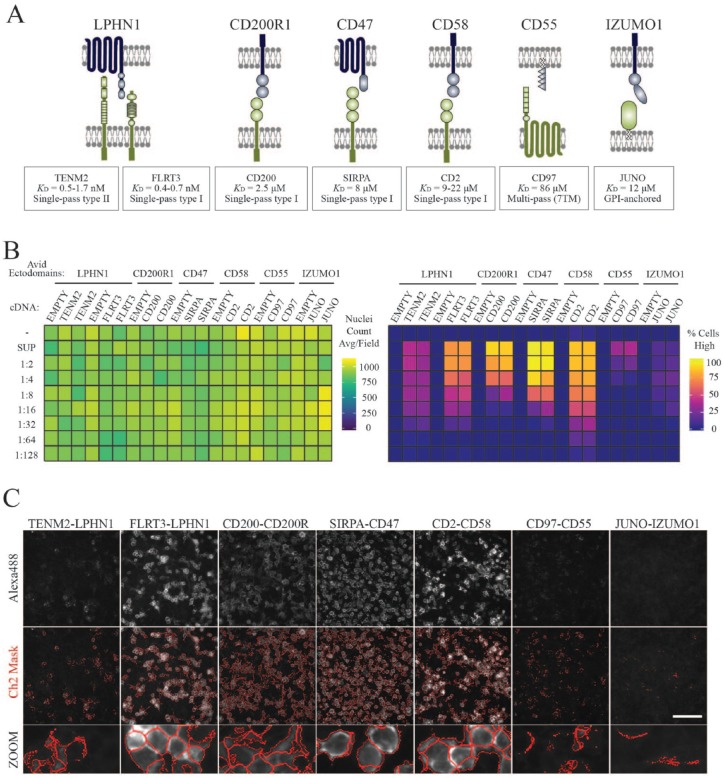
Automated image-based screening and analysis detected low-affinity cell surface interactions between different ligand–receptor classes. (**A**) Schematic of known extracellular interaction partners used as positive controls. The architectural receptor subclasses are indicated together with the reported affinity measurements (*K*_D_ values) for each interaction. (**B**) Ligand-bound cell populations were consistently detected across 384-well plates. Cells were transfected with 62.5 ng of cDNAs encoding full-length cell surface receptors using 0.15 µL of Lipofectamine per well. Avid recombinant ectodomains were incubated with transiently transfected cells as depicted in the 384-well layout. Concentrated supernatant (SUP) containing highly avid ectodomain probes was normalized and serially diluted down the plate (1:2 to 1:128). Cells incubated with complete media were used as negative control wells (–). Heatmaps depict the average number of nuclei per field and the percentage of cells that are characterized as having high Alexa488 fluorescence signals. (**C**) The application of target identification masks depends on the signal intensity of ligand–receptor interactions. Representative images for ligand–receptor pairs are shown with and without channel 2 target identification masks (red). Scale bar = 100 µm.

Using this image-based screening approach, we sought to verify a selection of low-affinity cell surface interactions between structurally varied and functionally diverse receptors. Seven known receptor–ligand pairs were chosen, with their previously reported *K*_D_ values depicted (**Fig. 3A**).^17,27–33^ This included interactions involving type I and type II single-pass cell surface receptor proteins, glycosylphosphatidylinositol (GPI)-anchored proteins, and multispanning membrane proteins. As some recombinant proteins are difficult to express in sufficient amounts for large-scale screening approaches, we also wanted to test the sensitivity of this assay to varying the concentrations of the soluble binding probe. Supernatants containing β-lactamase tagged ligands were concentrated, normalized, and serially diluted down the plate (**Fig. 3B, Suppl. Fig. S1A**). We found that we could confidently identify six out of seven receptor–ligand pairs, including the extremely weak (*K*_D_ > 80 µM) CD97–CD55 interaction (**Fig. 3B**). Images overlaid with target identification masks showed that weak Alexa488 signals cannot be readily distinguished from background fluorescence and therefore the Juno–Izumo interaction was difficult to detect using this technique (**Fig. 3C**). Even though cells remained attached across all wells, as established by the cell nuclei count, the percentage of cells with high-fluorescence signals in the Alexa488 channel decreased upon dilution of recombinant ectodomains. For optimal screening, we calculated the final concentration of avid probes to be in the range of ~10–20 µg/mL. To ensure that this assay was sufficiently robust at detecting ligand–receptor interactions, we manually inspected images across the plate and found that all wells consistently matched with image-based analysis measurements (**Suppl. Fig. S1B**).

**Figure 4.**
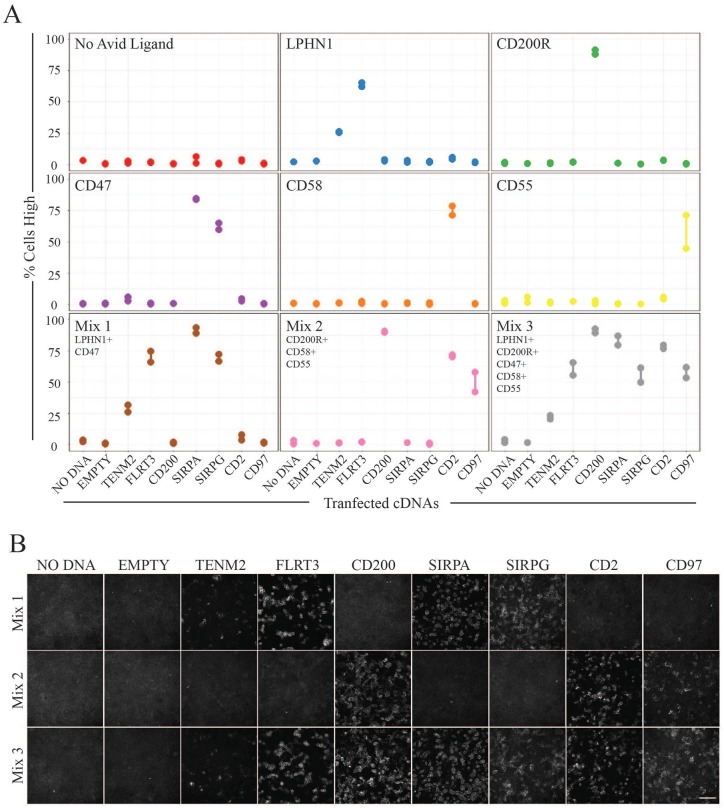
Pooling recombinant ectodomains increases screening throughput. (**A**) Ligand–receptor pairs are detected using pooled ectodomain probes. Cells were transfected with cDNAs encoding full-length cell surface receptors at 62.5 ng per well using 0.15 µL of Lipofectamine for each reaction. Mock-transfected cells and empty vector transfections were used as negative control wells. Recombinant ectodomains were concentrated using his-tag purifications and normalized with β-lactamase enzymatic activity. Recombinant protein ectodomains for LPHN1, CD200R1, CD47, CD58, and CD55 were incubated in wells individually and in pools (mixes 1–3). Measurements depict the percentage of cells bound by avid probes (% Cells High). (**B**) Representative Alexa488 channel images for the detection of ligand–receptor pairs using three distinct mixes of avid probes. Scale bar = 100 µm.

Depending on the number of plates that need to be screened, high-content screening can be resource-intensive in terms of materials, and may also require long acquisition times and large amounts of data storage. To try and increase the throughput of assay conditions, five recombinant ectodomains were purified, normalized, and combined in pools to test their ability to maintain receptor binding specificities under these conditions (**Fig. 4A, Suppl. Fig. S2A**). The same ligand–receptor pairings were tested as before, with the addition of the CD47–SIRPG interaction, which has been shown to have a binding affinity of ~23 µM.^34^ As shown previously, incubations with individual recombinant ectodomains led to high Alexa488 signals in a subpopulation of cells in accordance with the cell surface overexpression of their respective receptor pair (**Fig. 4A**). Importantly, specificity was maintained when avid ligands were pooled in three different mixes; combining two, three, and five distinct ectodomains, which was supported by manually inspecting images in the Alexa488 channel (**Fig. 4B, Suppl. Fig. S2B**). These results provided further confidence that the pooling of recombinant ectodomains can be used to increase the throughput of large-scale extracellular protein interaction screens.

### Large-Scale Screening of Ligand–Receptor Pairs Using a Cell Surface cDNA Expression Library

**Figure 5.**
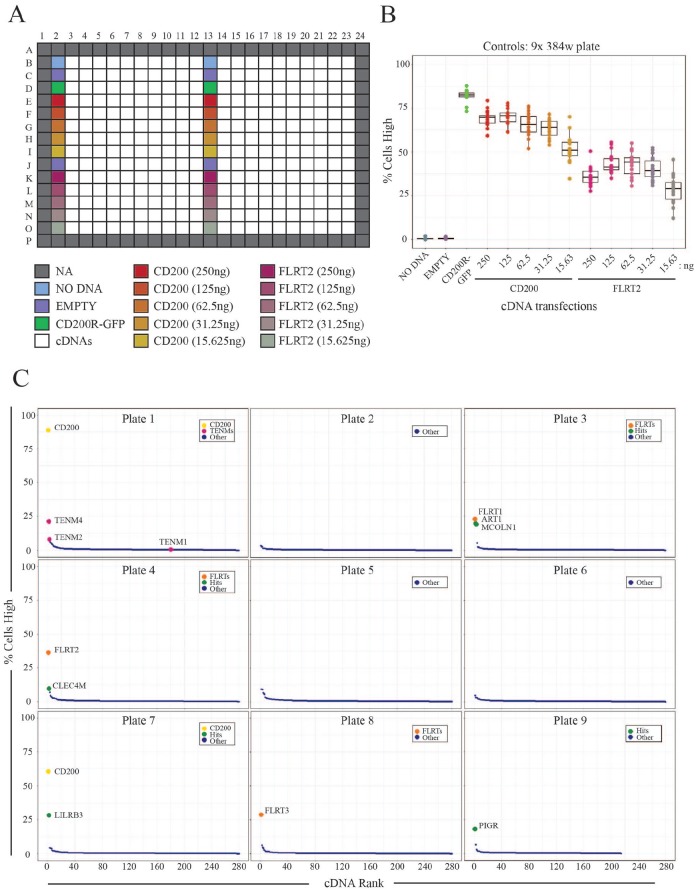
Systematic large-scale screening identified expected interactions. (**A**) Summary of 384-well templates for large-scale screening. Negative and positive control wells are located in columns 2 and 13. Positive control wells included a plasmid encoding full-length CD200R fused to green fluorescent protein (CD200R-GFP) and wells transfected with varying concentrations of plasmids containing full-length cDNAs encoding CD200 and FLRT2; these were used as controls since LPHN1 and CD200R are known to bind to FLRT family members (FLRTs) and CD200, respectively. Because of the plate edge effects, wells on the borders of plates were excluded from analysis (NA). (**B**) Positive control interactions were robustly and consistently detected across multiple 384-well plates. By measuring the percentage of cells with high Alexa488 fluorescence signals, negative and positive wells were clearly distinguished. (**C**) Large-scale image-based screening can identify extracellular cell surface interactions. Screening was performed using recombinant protein ectodomains for LPHN1, CD200R1, GPR64, and ZP2. Rank-ordered receptors bound by avid recombinant ectodomains across each of the nine 384-well plates are shown.

While we could employ image-based analysis to successfully identify ligand–receptor interactions in small focused studies, we next sought to determine if this approach could be used on a scale that would encompass the thousands of different proteins that have been identified at the surface of human cells.^3–5,7^ We therefore compiled a list of 2455 cDNAs encoding full-length human membrane-localized receptors (**Suppl. Table S1**). Most of the expression plasmids are untagged, but ~250 are C-terminally Myc-FLAG tagged. There is some redundancy within the plasmid library, with 54 genes represented by two or three different plasmids, although the majority of these were present as tagged and untagged forms. We have established that multiple probes can be used to identify interactions, and so we pooled four different proteins corresponding to the ectodomains CD200R, LPHN1, ZP2, and GPR64. CD200R and LPHN1 were included as positive controls since they have known ligands. ZP2 and GPR64 are both orphan receptors: ZP2 is localized to the zona pellucida that surrounds oocytes and is suggested to bind an undefined ligand expressed on sperm,^35^ and GPR64 belongs to the family of adhesion G-protein-coupled receptors (GPCRs) and is essential for male fertility.^36^ HEK293 cells were individually transfected with the library of 2455 cDNAs arrayed together with two columns of control cDNAs on nine 384-well plates; these control transfections were used to establish the Alexa488 fixed fluorescence threshold and the cellular response limit (**Fig. 5A**). Using a stringent cellular response limit, we could clearly distinguish negative and positive ligand-bound cell populations in control wells (**Fig. 5B**). During image acquisition, fields of view that had failed to autofocus were automatically rejected from downstream analysis. When this did occur, it was often a single out-of-focus image in a well, and as cell loss was not a problem, we could still effectively capture high numbers of nuclei from the three remaining fields of view for calculation of downstream measurements (**Suppl. Fig. S3A**). Nuclei counts were compared to assess variations in cell attachment across plates, and we found that large deviations were relatively uncommon (**Suppl. Fig. S3B**). Images were visually checked in wells characterized by having very low cell numbers, and these counts could be attributed to the poor application of segmentation algorithms, rather than through a loss of cell binding (**Suppl. Fig. S3C**). Across nine plates, known interacting partners for CD200R and LPHN1 were successfully identified (**Fig. 5C**). Two distinct cDNAs encoding both an untagged and tagged full-length CD200 were included in the library (plates 1 and 7), and both were robustly detected. LPHN1 is known to bind to paralogous family members of both the FLRTs and teneurins (TENMs), and all three FLRT paralogs were identified.^28–30^ Of the four TENM paralogs in the genome, we detected interactions with TENM2 and TENM4, and while TENM3 is a known ligand,^16^ cDNAs encoding this receptor were absent from the library. We identified five other plasmids that conferred positive binding signals that encoded the receptors ART1, MCOLN1, CLEC4M, LILRB3, and PIGR, and these were retested in independent confirmation assays with each individual binding probe. Both LILRB3 and PIGR overexpressing cells displayed cell surface staining (**Suppl. Fig. S3D**), but upon retesting were found to bind directly to Alexa488-labeled OX68, and are likely to interact with the constant domain of the labeled OX68 mouse IgG.^37^ Cells transfected with plasmids encoding CLEC4M exhibited weak cell surface staining (**Suppl. Fig. S3D**), but promiscuous binding of this receptor across multiple screens was observed, and as it is a C-type lectin family member, it may bind to ligands modified with carbohydrates. Although ART1 and MCOLN1 were detected as positive hits, the pattern of fluorescence did not localize to cell surfaces and instead appeared internalized (**Suppl. Fig. S3D**). We were unable to confirm the specificity of binding with individually tested recombinant ectodomains and therefore believe these receptors to be false-positive hits. In summary, the identification of the known binding partners for CD200R and LPHN1 demonstrated that this approach can be used to detect ligand–receptor interactions on a genome-wide scale, but no candidate ligands for either ZP2 or GPR64 ectodomains were identified.

## Discussion

Here we describe the development of a cell-based assay using large-scale transient transfections and HCI to detect extracellular binding events between ligand–receptor pairs. The assay is simple as only two readouts are measured: the total cell number (nuclei count) and the percentage of ligand-bound cells within the population. This means that images are acquired in two fluorescence channels, and because population statistics are recorded—rather than individual cell features—less data storage is needed. Analysis was performed simultaneously with image acquisition, and we carefully considered the number of fields imaged per well to reduce screening time. Due to the simplicity of image processing, this approach should be easily performed on most, if not all, HCI systems using either the supplied commercial software or open-source image analysis tools such as CellProfiler^38^ or Fiji.^39^

We demonstrated that established immunofluorescence protocols could be consistently applied across 384-well plates for the immunolabeling of recombinant ligands on cells. Paraformaldehyde is an appropriate fixative for this application since it preserved the epitope on the protein tag while maintaining the integrity of the plasma membrane. There is evidence that some surface receptors remain mobile within the membrane after fixation with 4% paraformaldehyde (e.g., GPI-anchored proteins),^40^ and supplementation with glutaradehyde^40^ or glyoxal^41^ could be considered for more complete/quicker fixation of cell membranes.

Here, we used recombinant ectodomains as the probe of choice, but as with other cell-based extracellular interaction assays, ligands such as peptides and pathogens could also be applied, as long as they could be easily detected through fluorescent labeling. Incubating recombinant proteins with living cells at 37 °C was sufficient to identify most of the known interactions tested in this assay; however, cells are still metabolically active, and it is known that binding of antibodies and ligands can promote receptor internalization. We did try incubations at lower temperatures (4 °C), but this led to some loss of cell adherence. Future refinements of the assay may include the addition of sodium azide, which can be used to prevent endocytosis, although the effect on GripTite HEK293 cell viability and adhesion would need to be established.

The cell-based approach described here to identify extracellular receptor–ligand interactions could have advantages over biochemical plate-based studies since we have shown that it can be used to identify interactions between architecturally diverse receptors. For example, we found that we could overexpress a multispanning transmembrane protein from the adhesion GPCR family (CD97) and confirmed binding with a known ligand. We established that we could scale the assay to include 2455 cell surface cDNAs and identify known interactions consistently across the library. Importantly, the number of false positives was low so that subsequent confirmation assays could be easily performed. To increase throughput, we found that ectodomain regions could be pooled, and this will help to save on resources since multiple interactions can be tested in a single experiment. However, we did find that large amounts of recombinant protein are required for this approach, much more than is required for assays involving the direct binding of recombinant ectodomains. This means that this assay may only be suitable for proteins that can be expressed and purified in large amounts when implemented at scale. Future refinements to reduce the amount of protein required could involve further miniaturization in 1536-well microplates, which are compatible with many HCI instruments.

The low-affinity interaction between the Juno–Izumo receptor–ligand pair was more difficult to detect in this assay as fluorescence signals were too weak for automated detection using the image analysis protocol described. It is possible that Juno’s transport is more tightly regulated by the cell, reducing the number of sites presented for Izumo binding at the cell surface. Differences in the levels of receptor expression or presentation on the plasma membrane are likely to be a source of variation that will affect the outcomes of screens and are a major limitation when compared with biochemical screens where ectodomain concentrations can be directly controlled. Even when receptors are expressed highly on cell surfaces, it could also be possible that the large pentamerization and detection tags on recombinant ectodomains impact on the identification of interactions through steric hindrance. Low expression of receptor pairs, steric hindrance, or simply an absence of a receptor-encoded cDNA within the library could account for false negatives, and may account for the lack of ZP2 and GPR64 novel binding events within the large-scale screen.

In conclusion, here we have described the optimization of a protocol for the transfection of cells in a 384-well plate format together with an HCI system to detect low-affinity extracellular protein interactions at a genome-wide scale.

## Supplemental Material

Supplemental_Material_for_HCI_extracellular_protein_interactions_by_Wood_et_al – Supplemental material for High-Content Imaging for Large-Scale Detection of Low-Affinity Extracellular Protein Interactions Click here for additional data file. Supplemental material, Supplemental_Material_for_HCI_extracellular_protein_interactions_by_Wood_et_al for High-Content Imaging for Large-Scale Detection of Low-Affinity Extracellular Protein Interactions by Laura Wood and Gavin J. Wright in SLAS Discovery

## Supplemental Material

Supplementary_Table_1_revised – Supplemental material for High-Content Imaging for Large-Scale Detection of Low-Affinity Extracellular Protein Interactions Click here for additional data file. Supplemental material, Supplementary_Table_1_revised for High-Content Imaging for Large-Scale Detection of Low-Affinity Extracellular Protein Interactions by Laura Wood and Gavin J. Wright in SLAS Discovery
