# Supplementary material for: High-Content Imaging for Large-Scale Detection of Low-Affinity Extracellular Protein Interactions
Source: SLAS Discov. 2019 Oct 3;24(10):987–99. doi: 10.1177/2472555219879053 (PMC6873222; doi:10.1177/2472555219879053)
Supplement: Supplemental_Material_for_HCI_extracellular_protein_interactions_by_Wood_et_al – Supplemental material for High-Content Imaging for Large-Scale Detection of Low-Affinity Extracellular Protein Interactions [file Supplemental_Material_for_HCI_extracellular_protein_interactions_by_Wood_et_al.pdf]

Supplemental material

High content imaging for large-scale detection of low affinity extracellular protein interactions

Laura Wood[a] and Gavin J. Wright[a]

[a] Cell Surface Signalling Laboratory.

Wellcome Trust Sanger Institute,

Cambridge,

CB10 1SA,

United Kingdom.

Corresponding author:

Gavin J. Wright

Cell Surface Signalling Laboratory

Wellcome Trust Sanger Institute

Cambridge CB10 1SA

United Kingdom

Email: gw2@sanger.ac.uk Tel: +44 (0) 1223 496852

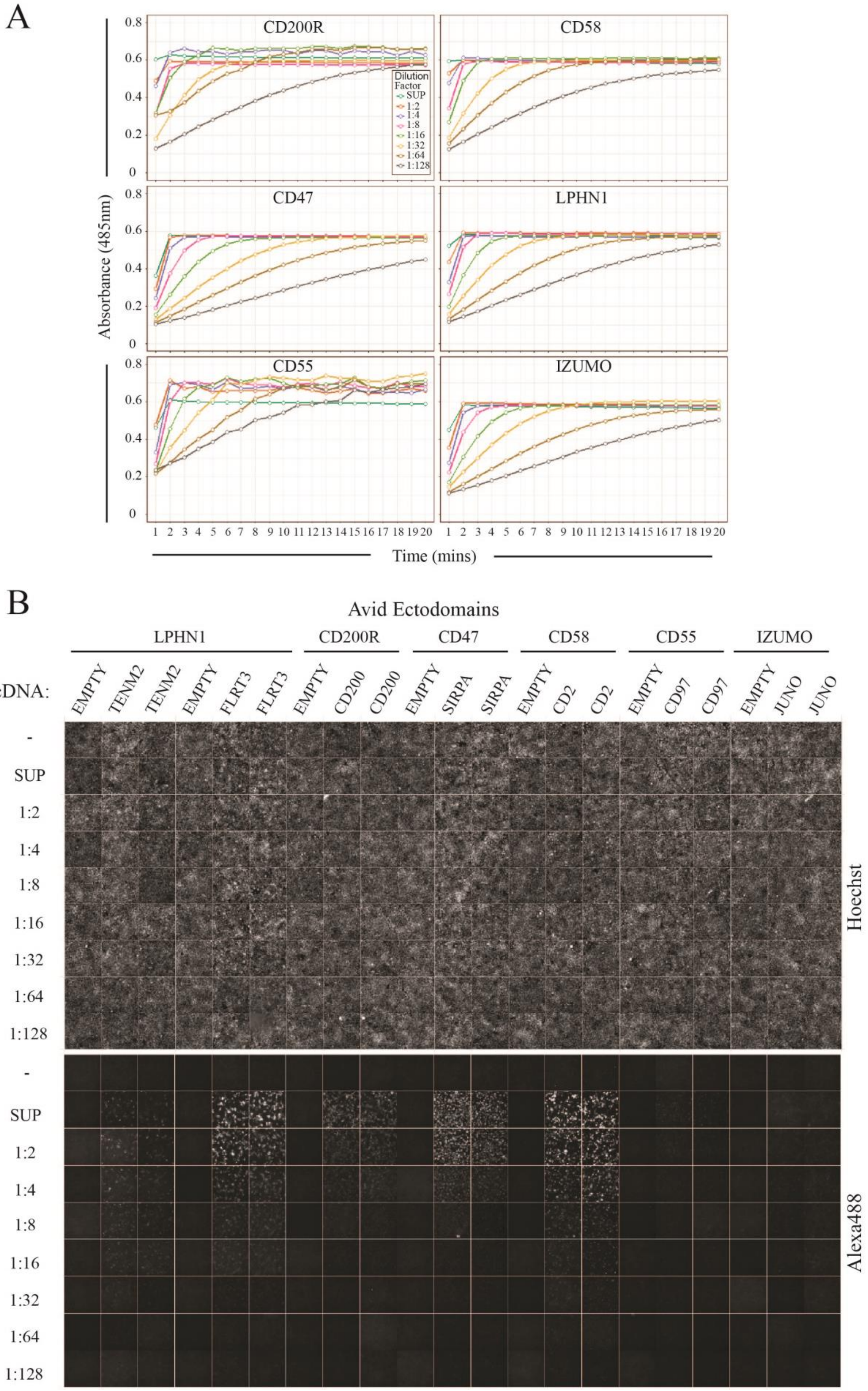
A
CD200R
CD58
CD47
LPHN1
CD55
IZUMO
Dilution Factor
SUP
1:2
1:4
1:8
1:16
1:32
1:64
1:128
Absorbance (485nm)
Time (mins)
B
Avid Ectodomains
LPHN1
CD200R
CD47
CD58
CD55
IZUMO
cDNA:
EMPTY
TENM2
TENM2
EMPTY
FLRT3
FLRT3
EMPTY
CD200
CD200
EMPTY
SIRPA
SIRPA
EMPTY
CD2
CD2
EMPTY
CD97
CD97
EMPTY
JUNO
JUNO
-
SUP
1:2
1:4
1:8
1:16
1:32
1:64
1:128
Hoechst
Alexa488

Figure S1. Recombinant protein ectodomains were used to detect extracellular ligand-receptor interactions across 384-well plates. A. Pentameric β-lactamase-tagged ectodomains were quantified and normalised using nitrocefin hydrolysis. Neat supernatant (SUP) containing the recombinant proteins were spin-concentrated so that all the nitrocefin substrate, as measured by absorbance at 485nm, was hydrolysed after five to ten minutes when diluted 1 in 32. A serial dilution of the final concentrated supernatant is shown for each probe. **B.** Manual inspection of acquired images for Hoechst-33342 and OX68-Alexa488 stained cells. Cells were transfected with cDNAs encoding full-length human cell surface receptors and incubated with serially diluted supernatant (SUP) containing recombinant protein ectodomains as indicated. Negative control wells (-) were treated with complete media.

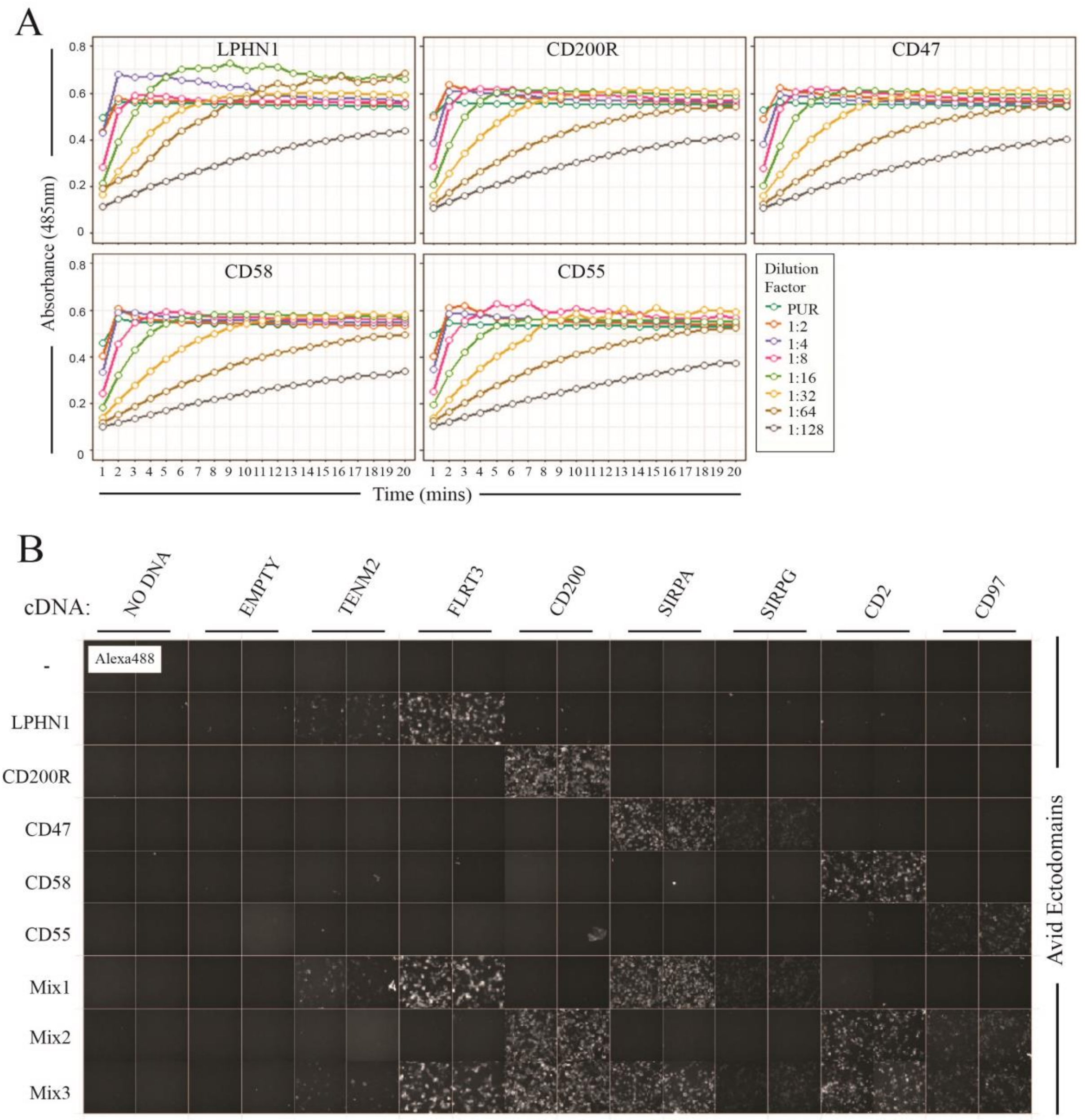

Figure S2. Purified recombinant probes can be pooled to detect multiple ligand-receptor interactions in a single experiment. A. Pentameric recombinant ectodomains were purified using his-tag purification protocols and normalised using beta-lactamase enzymatic activity. Purified proteins (PUR) were normalised to a concentration where all the nitrocefin substrate, as measured by absorbance at 485nm, was hydrolysed after five to ten minutes when diluted 1 in 32. **B.** A montage of Alexa488 signals detected across a 384-well plate. Cells were transfected with cDNAs and incubated with individual or mixed recombinant protein ectodomains. Mix1= LPHN1 and CD47, Mix2= CD200R1, CD58 and CD55, Mix 3= all five ectodomains. Negative control wells (-) were treated with complete media.

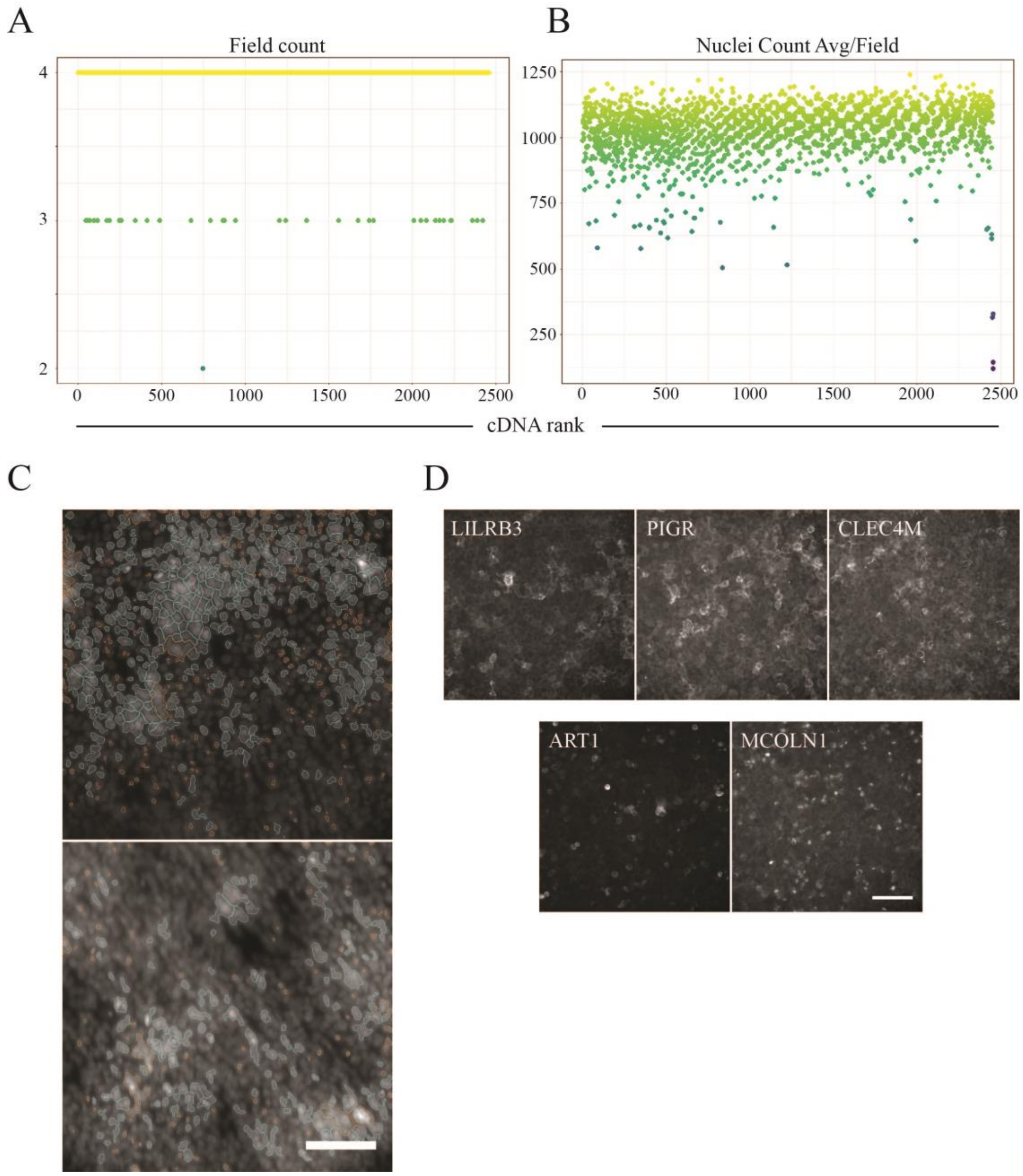

Figure S3. Detection of cell count remains relatively consistent across large-scale screens. A. Failed image autofocusing reduces the number of images available for analysis. Out-of-focus images were rejected from downstream processing, but this was a relatively uncommon occurrence, with usually three or four fields available in each well for analysis. **B.** Nuclei can be segmented consistently across large-scale screens. The average number of segmented nuclei per field ordered based on ranked cDNAs encoding cell surface receptors bound by avid recombinant ectodomains. **C.** Low nuclei count is seen in wells with poorly segmented nuclei and often this does not reflect a complete loss of cell attachment. **D.** False-positive hits were identified in large-scale cell surface interaction screens. While cells overexpressing LILRB3, PIGR and CLEC4M displayed cell surface staining, ART1 and MCOLN1 signals localised internally. Scale bar: 100 µm.
